# An integrated view of immunological tolerance

**DOI:** 10.1111/sji.13207

**Published:** 2022-07-26

**Authors:** Peter A. Bretscher

**Affiliations:** ^1^ Department of Biochemistry, Microbiology and Immunology, College of Medicine University of Saskatchewan Saskatoon Saskatchewan Canada

## Abstract

I summarize how diverse mechanisms may contribute to self‐tolerance, and how an appreciation of their interdependence is necessary to understand how they collectively provide the imperfect degree of tolerance achieved. I suggest that the Quorum Hypothesis of Lymphocyte Activation incorporates one means of achieving peripheral tolerance. This hypothesis posits that the activation of all lymphocytes requires their antigen‐mediated interaction with other lymphocytes, whereas single lymphocytes are inactivated by antigen. Mature lymphocytes, specific for a peripheral self‐antigen, are eliminated as generated, one or a very few at a time, due to the first presence of the antigen early in development and continuously thereafter. There are limits to the circumstances under which this mechanism leads to tolerance. For example, if most self‐specific lymphocytes were not eliminated by the mechanism of central tolerance, the emigration of anti‐self lymphocytes, from primary lymphoid organs, would occur at so high a rate that the quorum mechanism would fail to eliminate but lead to their activation. Evolutionary considerations lead me to suggest that the thymic expression of certain self‐antigens, via such transcription factors as the autoimmune response element, allows more rapid immune responses against invaders without resulting in autoimmunity. This is clearly evolutionarily advantageous. It has been proposed that novel self‐antigens may be expressed at certain stages of life, such as adolescence. I discuss how tolerance to ‘adolescent antigens’ may be achieved, and to a special class of ‘newly arising self‐antigens’, namely idiotypic epitopes of the antigen‐specific receptors of B and T lymphocytes.

## 1 INTRODUTION

This article has a significant developmental history. I recently published a Discussion Forum article in this journal, entitled ‘The Historical Postulate: Is it the basis, at the level of the system, for Self‐Nonself Discrimination?’.^1^ This drew a response from Colin Anderson, entitled ‘The historical postulate is not the basis of self‐nonself discrimination: A response to Bretscher’s proposal’.^2^ This article is the third version of my response to Anderson’s article. The first two versions of my response led to signed reviews by Colin Anderson and Alex Corthay. The reviews by both individuals were extensive and considered. Their reviews, and my resubmissions, constitute unpublished discussions. It is unique in my experience that a submitted manuscript receives such considered attention. I am indebted to both reviewers. Corthay recommended that my revised manuscript should be readily understandable on its own, without requiring the reader to consult my original article and Anderson’s response to it. I saw the wisdom of this advice and acted upon it. Nevertheless, it left me feeling somewhat uneasy. Firstly, I am often taking advantage of the considered comments of the reviewers without giving them explicit credit. To do so would make this account less fluent. Secondly, I do not exclusively focus on the differences in opinion still existing between Dr Anderson and myself, with an attempt to justify mine. Such a focus would be natural in a discussion forum. I emphasize ideas that arose in response to the reviewers’ comments. I also address some issues where Anderson and I have different opinions. I stress again that the reviewers contributed to the final product, even if they may not agree with some of it. I express my appreciation to both.

## 2 EVIDENCE AND IDEAS AT DIFFERENT LEVELS

The science of genetics at one time provided only a description of the laws of inheritance at the level of the system. Mendel’s laws do not refer to cells or molecules, nor do the rules governing genetic linkage. The origin of molecular biology was in large measure inspired by the wish to understand how these precise laws at the level of the system are realized in molecular terms. This explanation provided a criterion, and a most important criterion, for the molecular framework to be considered satisfactory. Genetics inspired and guided the central ideas that became molecular biology.

My awareness of the importance of different levels of analysis has surely been heightened by studying physics. Thermodynamics consists of laws that hold independently of the nature of the materials involved. A major and intuitive question in biology is: how can such complex and organized systems, as animals are, spontaneously arise in nature? Thermodynamic considerations led to the understanding of the central role of the sun in supporting photosynthesis, the growth of plants and so the direct or indirect source of food, that allow animals to exist. This is an example of how a question can be raised at the level of the system, and laws discovered at this level can be employed to guide answers at a more detailed level.

Immunology is a science in which there are observations and considerations made at the level of the system, and at the levels of cells and of molecules. The significance given to a molecular observation by a researcher usually depends upon the framework the researcher consciously or unconsciously entertains at the level of the system. Valid ideas at the level of the system bring more detailed and diverse observations together, often at the molecular level, and provide a context for understanding the significance of the detailed observations as to how the system functions. Invalid frameworks at the level of the system will usually result in an invalid significance being attributed to more detailed observations.

I engaged in conversations with immunologists when I first entered the field over 50 years ago. They suggested to me there was a myriad of mechanisms, pertinent to different self‐antigens, that together accounted for self‐tolerance. I thought then, and think now, this view implausible, especially from an evolutionary perspective. At the point in evolution, when an innate defence mechanism gave rise to an adaptive system with wide receptor diversity, and so the ability to respond to the unknown, mechanisms had to coevolve to ensure unresponsiveness towards self. This must mean that there was initially one (or a very few) mechanisms ensuring tolerance of self. A myriad of mechanisms is implausible. I thought it plausible that there must be a property *shared by all self‐antigens* that is relied upon in ensuring self‐tolerance. The Historical Postulate, that states tolerance to self‐antigens requires their early presence in development^3^ and their continuous presence thereafter,^4^ seemed to describe the obvious, and the only, possibility, as had already been proposed by Burnet^3^ and by Lederberg.^4^

## 3 THE RELATIONSHIP BETWEEN CONCEPTS AND OBSERVATIONS AT DIFFERENT LEVELS

Ideas and observations at different levels can be either consistent or inconsistent with one another. They are not competitive in the most direct sense. A more detailed description can be consistent/inconsistent with a broader‐level description; a broader‐level description can be consistent with several more detailed descriptions. For example, The Historical Postulate is consistent with different cellular mechanisms, as we shall see, and these different cellular mechanisms are each consistent, I shall argue, with The Historical Postulate.

The Historical Postulate is an idea at the level of the system, as reflected in the title of my first paper. Its meaning is defined in the previous section. The postulate addresses what circumstances determine whether an antigen induces an immune response or ablates/prevents the potential of such a response. What are the currently alternative ideas *at the level of the system* for *mature lymphocytes*? These are primarily the highly popular *pathogen‐associated molecular pattern (PAMP)* and the *danger‐associated molecular pattern (DAMP)* models, espoused primarily by Janeway^5^, ^6^ and Matzinger.^7^, ^8^ (This is a simplification, as I indicate below, but at least allows a clear statement to be made, and a framework to be outlined.) I now give a brief account of how these different ideas at the level of the system arose, and my view as to why these models are incompatible with the postulate, so that readers can follow the thread of this manuscript without reading the preceding papers.

## 4 WHAT DETERMINES, AT THE LEVEL OF THE SYSTEM, WHETHER ANTIGEN INDUCES IMMUNITY OR WHETHER ANTIGEN OBLITERATES THIS POTENTIAL?

### 4.1 Recognition of the problem

Paul Ehrlich immunized a goat with red blood cells of another goat. The antibody reacted with the donor goat’s red cells but not with the red cells of the immunized goat. The red cells of the two goats were clearly different. Ehrlich realized that production of antibody against the immunized goat’s own red cells would be devastating. He imagined that there must be a way of preventing this.^9^ Immunologists paid more attention to Ehrlich’s concerns with the discovery in the late 1930s of autoimmune haemolytic anaemia.^10^ Antibodies to the patients’ own red cells could have devastating consequences.

### 4.2 The historical postulate

Burnet and Fenner in the late 1940s proposed how it may be possible for the immune system to respond against foreign, non‐self antigens but not against the self‐antigens of the body to which the immune system belongs. They proposed the presence of self‐antigens early in development destroyed the immune system’s ability to respond against them.^3^ Medawar,^11^ Hasek^12^ and their colleagues showed that the exposure of developing foetuses to a foreign antigen ablated the ability of the animal, as a young adult, to make an immune response on challenge with the antigen. These observations were taken as supporting Burnet and Fenner’s conjecture.

The periodic administration of antigen was found to be required to maintain the unresponsive state.^13^ Lederberg concluded that new lymphocytes are continuously generated throughout life. That is why the antigen is required to maintain tolerance.^4^ Thus, tolerance to a self‐antigen likely requires the presence of this antigen early in development, that is, in the life history of the animal, and its continuous presence thereafter. I refer to this proposal as The Historical Postulate.

### 4.3 Central and peripheral tolerance

It is recognized that most anti‐self lymphocytes are eliminated in primary lymphoid organs, as generated, if the self‐antigen is sufficiently present.^14^ This is in accord with the Historical Postulate and Lederberg’s proposal of 1959. He envisaged that immature lymphocytes are silenced on contact with antigen; if not silenced within a day or so, they differentiate into a mature state, where their interaction with antigen leads to their activation.^4^ This process of elimination of most anti‐self lymphocytes is referred to as central tolerance. It became later recognized that some self‐antigens, such as insulin,^15^ are not present in primary lymphoid organs at a level to eliminate all their corresponding precursor cells. Lymphocytes specific for these peripheral self‐ and foreign antigens emigrate from the primary lymphoid organs into the periphery. It is now generally accepted, in contrast to what Lederberg supposed, that these mature lymphocytes can be either inactivated or activated by antigen.^16^ It is natural to assume that, under the best of circumstances, peripheral self‐antigens inactivate their corresponding lymphocytes and foreign antigens activate theirs. Such a process would lead to a state referred to as peripheral tolerance. Most models of organ‐specific autoimmunity envisage that such autoimmunity arises from a failure of peripheral tolerance.

## 5 JANEWAY’S AND MATZINGER’S PROPOSALS ARE INCONSISTENT WITH THE HISTORICAL POSTULATE

Janeway, in 1989, addressed how antigen might interact with mature T cells to cause their activation.^5^ He accepted evidence that antigen required activated helper T cells to activate most B cells and, in the absence of this help, antigen inactivated the B cell.^17^, ^18^ It was known that the activation of CD8 T cells could be facilitated by activated CD4 T cells.^19^ It was later shown, by Guerder and Matzinger, that, in the absence of help, antigen can inactivate CD8 T cells.^20^ Although Janeway addressed what the requirements were to activate both CD4 and CD8 T cells, I centre my discussion of his considerations around CD4 T cells, as only this seems germane to current debate. In addition, I must point out that Janeway and Medzhitov did not squarely face the problem of peripheral tolerance, in the sense they suggested peripheral antigens did not exist. My recent rereading, thanks to Dr Anderson, leads me to conclude that they suggested there was no need for peripheral tolerance at the level of CD4 T cells, as central tolerance was sufficient.^6^, ^21^ They say: ‘Self‐peptides expressed and presented by APCs are not recognized as nonself, because T cells specific for these peptides are eliminated during negative selection in the thymus. Thus negative selection and microbial induction of costimulatory molecules together ensure that the adaptive immune response is generated against infecting pathogens but not against self‐antigens’.^6^ I am honestly puzzled by this stance. It makes an explanation of organ‐specific immunity problematic, as well as many other observations I shall discuss.

Janeway came to the above proposal on the role of microbial components in activating CD4 T cells in view of a generalization. Many purified proteins are not immunogenic unless given in an adjuvant containing microbial components, such as complete Freund’s Adjuvant (CFA). He suggested the interaction of a pattern recognition receptor (PRR) with a PAMP was necessary to stimulate the antigen‐presenting cell (APC) to express the requisite costimulatory (CoS) molecule for CD4 T‐cell activation. Five years later, Matzinger pointed out that vertebrate, PAMP‐free foreign grafts are immunogenic, a fact paradoxical in the context of Janeway’s proposal. Based on this and other grounds, she proposed that the requisite costimulatory molecule would be expressed only under dangerous circumstances, that is, when a DAMP is expressed.^7^ Matzinger also envisaged that, in the absence of the requisite CoS signal, antigen would inactivate the CD4 T cells.^7^ In the context of current ideas, most believe a DAMP/PAMP signal is required for antigen to activate CD4 T cells and, in the absence of such a signal, antigen can inactivate the CD4 T cell. This is the view that I consider here and that I suggest is implausible.

Activated CD4 T cells appear to be required for antigen to activate most B and CD8 T cells, and antigen inactivates these cells in the absence of help, as outlined above. The circumstances determining whether antigen inactivates/activates CD4 T cells thus determine whether the potential of an immune response is ablated or realized. Thus, Janeway’s model implies that the immune system does not discriminate self from non‐self but rather the non‐infectious from the infectious, and Matzinger antigen associated with non‐dangerous from antigen associated with dangerous circumstances. (Janeway, I think, may have been uneasy about this; he referred for years to his idea as the immune system discriminating non‐infectious self from infectious non‐self, which seems confusing.^22^ It leaves open the question of how the immune system responds to non‐infectious, non‐self, as I discuss below). Janeway’s and Matzinger’s models have several features in common. When I want to refer to common features, I collectively refer to them as the PAMP/DAMP Model. This does not mean to imply that they are one and the same model.

The PAMP/DAMP Model is clearly distinct from models that propose how antigen interacts differently with lymphocytes to activate and inactivate them to achieve self‐nonself discrimination. Note that all models as understood today imply that, if the condition explicitly described is not satisfied, the alternative is realized. In the PAMP/DAMP Model, failure to satisfy the conditions for activating CD4 T cells results in antigen inactivating CD4 T cells. In models designed to be in accord with The Historical Postulate, an antigen, whose presence is not described by the postulate, is predicted to be immunogenic, if having the intrinsic properties required to be antigenic, mainly size and being proteinaceous. Thus, the postulate addresses not just the basis of self‐tolerance but also of self‐nonself discrimination.

## 6 AN INITIAL COMMENT ON THE DIFFERENCES OF ANDERSON’S AND MY PERSPECTIVE

The title of Anderson’s reply to my article was: ‘The historical postulate is not the basis of self‐nonself discrimination: A response to Bretscher’s proposal^[^ ^2]^ ^’^. Anderson implied by the title and content of his reply that The Historical Postulate is not uniquely at the top of the *hierarchy of concepts* central to understanding tolerance. Rather, he suggests, timing (during the development/life of the animal) of antigen exposure and the locations of the self‐antigen together constitute this pinnacle. If the self‐antigen is sufficiently present in primary lymphoid organs, central tolerance is established. If not, the unresponsiveness to self must rely on the mechanism(s) of peripheral tolerance. I think we are to a considerable extent talking at cross purposes, though I gather that Anderson may disagree. I made the case that The Historical Postulate is pertinent *at the level of the system* in considering mechanisms by which *self‐antigens* obliterate rather than realize the potential of responses against them. The alternatives *at the level of the system*, and in the context of peripheral tolerance, are that it is the antigen, in the absence of PAMP/DAMP signals, that ensures obliteration. I never used the word hierarchy in my analysis. I of course recognize the importance of the location of self‐antigens in determining whether tolerance is achieved by a central or a peripheral mechanism. However, location of antigen is hardly a criterion at the level of the system that explains how immune responses are respectively ablated or realized by self‐ and foreign antigens.

Anderson also suggests that the Danger Model is not really that inconsistent with the postulate. I will return to this issue later.

## 7 THE 1970 TWO SIGNAL MODEL

Central to discussions on immunological tolerance is The Two Signal Model of lymphocyte activation that Cohn and I proposed in 1970.^23^ I summarize, to provide context, its essential elements, as well as considerations and evidence that make me still favour it today.^24^

### 7.1 The model

The model was proposed to account for several observations then regarded as paradoxical and to incorporate a mechanism of peripheral tolerance consistent with The Historical Postulate. I address here just the mechanism.

The essence of our ideas can be expressed as the one lymphocyte/multiple lymphocyte model for the antigen mediated inactivation/activation of lymphocytes. Consider a peripheral antigen such as insulin and present in the periphery, in accord with The Historical Postulate, before any lymphocytes are generated. The first insulin‐specific, mature lymphocyte will be a loner and so inactivated. Insulin‐specific lymphocytes will be inactivated as generated, one or a few at a time. Lymphocytes specific for a foreign antigen, F, will accumulate in its absence; once F impinges upon the immune system, it can mediate the lymphocyte cooperation needed to initiate an immune response.

We developed a notation to encapsulate our proposal more fully. We suggested that when antigen interacts with a lymphocyte’s antigen‐specific receptors, signal 1 is generated; when generated alone for a few days the lymphocyte is inactivated. The activation of a target lymphocyte also requires the generation of signal 1, but in addition signal 2, its initiation occurring when an activated ‘helper lymphocyte’ binds antigen.

Corthay in one review asked me to clarify whether the two‐signal model bears on whether mature lymphocytes, as they emigrate from primary lymphoid organs, go through a Lederberg‐like state where they can only be inactivated by antigen. Mature lymphocytes, present in primary lymphoid organs, can be activated by antigen under appropriate circumstances. Thus, Claman showed there to be synergy in the antibody response to sheep red blood cells (SRBC) when irradiated mice were reconstituted with *both* bone marrow cells and thymocytes.^25^ This and other examples show that there are activatable lymphocytes in primary lymphoid organs. It therefore seems unlikely that recent emigrants go through a stage where they can only be inactivated by antigen. One feature of our model is relevant to this point. We could explain how the impingement of a foreign antigen, that cross‐reacts with a peripheral self‐antigen, can induce cross‐reactive lymphocytes that are not induced by the peripheral self‐antigen, as there are so few lymphocytes specific for the peripheral self‐antigen.^23^, ^26^ An example is the induction of heart‐specific lymphocytes upon infection by group A streptococci.^27^ The generation of this autoimmunity shows activatable lymphocytes specific for peripheral heart antigens exist. We infer that recent lymphocyte emigrants do not go through a phase where they can only be inactivated by the peripheral self‐antigen.

### 7.2 Some evidence supports The Two Signal Model

The activation and inactivation of most B cells and CD8 T cells are in accord with the model, as indicated and referenced above: their activation requires activated helper CD4 T cells and, in the absence of such help, antigen inactivates the B and CD8 T cells.^24^ These generalizations point to the central role of CD4 T helper cells in controlling whether immune responses are generated, or this potential is obliterated.

### 7.3 The principle of non‐interference

I consider this principle to be at the heart of how peripheral tolerance is realized. It encapsulates the idea that the inactivation by a peripheral self‐antigen, pS, of pS‐specific CD4 T cells, is not interfered with by an immune response to a non‐cross‐reacting foreign antigen, F, in the presence of F. Such a response would involve the generation of activated, F‐specific, CD4 T cells. In practice, as discussed below, there are diverse on‐going immune responses in immunocompetent vertebrates^28^, ^29^ and so, if this principle did not hold, peripheral tolerance could not be efficiently maintained, unless these processes were somehow separated by the location where they occur, an unlikely possibility as a generality. I return later to the issue of whether danger can be localized.

I think it helpful to first consider some of our findings that address expectations based upon the principle. My laboratory had already experimentally examined whether the activation of CD4 T cells required/is facilitated by CD4 T‐cell cooperation at the time Janeway first made his proposal.^5^ The PAMP/DAMP Model implies that single CD4 T cells can be activated under appropriate circumstances. We had found that the presence of Q‐specific CD4 T cells could facilitate the activation of R‐specific CD4 T cells, where R and Q were chosen not to cross‐react, in the presence of the conjugate R‐Q, but not in the presence of R and Q as separate molecules.^30^ This finding was not accidental but anticipated. We had thought CD4 T‐cell cooperation between R‐ and Q‐specific CD4 T cells must involve the recognition of linked epitopes (R‐Q) and should not be mediated by the recognition of unlinked epitopes (R and Q). Consider the following consequences if the latter situation held. The inactivation by a peripheral antigen, pS, of a single, pS‐specific CD4 T cell, could be interfered with by a CD4 T‐cell response to an irrelevant foreign antigen, F, in the presence of F. This circumstance would *not* allow The Principle of Non‐Interference to be realized. I later formulated this idea as a ‘formal’ principle to express why I found a feature of the PAMP/DAMP Model implausible. As the PAMP/DAMP signal is not generated following antigen recognition by lymphocytes, and is non‐specific, a response against F could interfere with the inactivation of a single CD4 T cell by pS. Despite our finding of a requirement for linked recognition, it was initially difficult to imagine how this requirement could be ensured.

Given that CD4 T cells recognize antigen in the context of class II MHC molecules, the CD4 T‐cell interaction must be mediated by an *antigen‐presenting cell* (APC) able to present antigens in the context of class II MHC molecules. It was well known that most APC will present both R and Q to their respective CD4 T cells, as assessed by a variety of criteria, when exposed to R and Q as separate molecules. I came slowly to the conclusion that only an antigen‐specific B cell could mediate CD4 T‐cell cooperation involving the recognition of linked epitopes. An R‐specific B cell would only present R when exposed to both R and Q unlinked, and a Q‐specific B cell would under similar conditions only present Q; however, both Q‐ and R‐specific B cells would present both antigens if exposed to the R‐Q conjugate. Therefore, it seemed likely an antigen‐specific B cell must mediate the CD4 T‐cell interaction.^31^

### 7.4 Evidence for the involvement of B cells in the activation of CD4 T cells

I have naturally reviewed elsewhere the evidence for such B cell involvement.^24^ I just describe one study as it is so physiologically relevant. Janeway and colleagues reported that activated CD4 T cells could be raised in mice to the self‐antigen, mouse cytochrome C (MCC). They could *not* generate such Th cells by immunizing with MCC given in CFA. To do so, they had also to give activated MCC‐specific B cells at the time of immunization with MCC in CFA. These activated B cells were raised in a donor mouse, immunized with human cytochrome, in a way that most likely involved the action of specific CD4 T helper cells.^32^ This experiment is paradoxical in the context of Janeway’s PAMP Model. It provides very strong evidence for a model in which CD4 T cell activation requires CD4 T‐cell collaboration mediated by an antigen‐specific B cell.

## 8 WHERE I THINK ANDERSON AND I AGREE, INCLUDING A SITUATION THAT ‘VIOLATES’ THE POSTULATE

This paper started out to discuss Anderson’s response to my first article on the postulate. Some of the content in the preceding pages has been motivated by Anderson’s comments in his paper, and his and Corthay’s comments as reviewers of my paper, submitted as a response to Anderson’s. I summarize what I think is Anderson’s^2^ and my shared view.^1^ This clears the ground for explaining why I think the different ways we describe our common view is significant. It also provides a context for a consideration of where our views differ.

We both primarily address the mechanisms of peripheral tolerance, agreeing with the main conclusions, outlined above, concerning the major role of central tolerance in preventing responses against self. I, of course, stress that the mechanism involved is consistent with The Historical Postulate.

Anderson and I agree with the plausibility of The Quorum Proposal, namely that a quorum of antigen‐specific lymphocytes is required for an optimal dose of antigen to activate lymphocytes and so initiate an immune response.^26^ In the absence of quorum, antigen inactivates lymphocytes. This proposal is a more contemporary formulation of our 1970 proposal. The Quorum Proposal explains peripheral tolerance in a manner consistent with The Historical Postulate, as outlined in our articles.

Anderson and I agree at the cellular/molecular level on how the observations discussed add up to provide an explanation of peripheral self‐nonself discrimination. This includes a situation where, strictly speaking, the postulate is shown not to hold, as originally stated. These concern studies with T‐cell receptor (TcR) transgenic mice that I outlined in my paper,^1^ where the transgenic TcR recognizes a peripheral antigen in the context of a class II MHC restriction element. These mice are found to be autoimmune against the peripheral antigen. I had suggested in my original paper on the postulate that the anticipated, rapid generation of antigen‐specific CD4 T cells in the thymus of these TcR transgenic mice, and the high rate of their emigration into the periphery, was likely orders of magnitude greater than the rate of emigration of lymphocytes specific for natural, peripheral antigens, such as insulin. I suggested that this could explain why autoimmunity arises, even though the peripheral self‐antigen is most likely present before *any* lymphocytes are generated.

Cohn and I had discussed in our 1970 paper^23^ the importance of the relationship between the time it took for different processes, involved in the activation and inactivation of lymphocytes, to have irreversible consequences. Consider the antigen‐dependent inactivation of a lymphocyte. We pointed out that the time to achieve irreversible inactivation of the lymphocyte must be at least a day or so, as it would take time, at least a day, for antigen to begin activating the cooperating lymphocytes, if any were present. If antigen could irreversibly inactivate a lymphocyte in a short time, say an hour, before the antigen could mediate any interaction between lymphocytes, antigen would never be able to activate any lymphocytes. As we proposed that the activation of CD4 T cells requires CD4 T‐cell collaboration, whereas antigen could inactivate single CD4 T cells, we anticipated the time to achieve significant T helper cell activation would depend on the frequency of the CD4 T cells. In the case of the studies just described, involving TcR transgenic mice, the rate at which antigen‐specific CD4 T cells emigrate from the thymus is orders of magnitude greater than the rate of lymphocytes specific for peripheral antigens, such as insulin. Consequently, it is natural to suppose activated Th cells can be generated before the CD4 T cells are irreversibly inactivated by antigen. In this case, it is not surprising that the postulate fails; in fact, the circumstances of the failure support The Quorum Hypothesis! Anderson agreed with this explanation for why autoimmunity arose and agreed it did not ‘violate’ the postulate in a serious sense.

## 9 SUMMARY OF ONE AREA OF DISAGREEMENT WITH ANDERSON AND THE RATIONALE FOR MY POSITION

In my original paper, making the case for the centrality of the postulate, I discussed several experimental findings that appeared at face value to violate the postulate.^1^ I concentrate here on just two studies, beyond the studies just outlined with TcR transgenic mice, as I think a discussion of these two is sufficient to bring up the important considerations. Anderson brought up a further study in his response that I do not discuss here.

### 9.1 Autoimmune response element (AIRE)‐deficient mice and the Anderson study

Mice, engineered to be deficient in the autoimmune response element, AIRE‐deficient mice, are autoimmune.^33^ The AIRE gene codes for a transcription factor that controls the expression of a variety of antigens that were once thought of as ‘peripheral antigens’, including insulin; in other words, these antigens were previously thought to be only expressed in the periphery. Evidence suggests that the thymic expression of these antigens contributes to central tolerance but, as in the case of insulin, this presence is, in at least some cases, insufficient to eliminate *all* the T cells specific for the peripheral antigen. Thus, some AIRE‐sufficient mice develop autoimmune diabetes, associated with activated, insulin‐specific T cells.^34^ Anderson thinks the explanation of the autoimmunity associated with AIRE‐deficiency is ‘more problematic for the historical postulate’ than the studies outlined above with TcR transgenic mice. He says this because the self‐antigens, whose expression in the thymus is normally controlled by AIRE, are also expressed extra‐thymically early in development. The increase in generation of specific T cells is modest, as a consequence of AIRE‐deficiency and, so he argues, the autoimmunity in AIRE‐deficient mice violates The Historical Postulate.^2^ (The peripheral antigens recognized by the transgenic receptor are also believed to be expressed extra‐thymically early in development). I will argue that these observations do not violate the postulate, in the sense that the postulate holds for actual *peripheral self‐antigens under normal circumstances*. However, I also need to summarize some of Anderson’s own observations,^35^, ^36^ discussed in my previous article, to respond to these comments.

Anderson and colleagues implanted foreign grafts in an internal site, such as under the kidney capsule, into immuno‐incompetent mice. They reconstituted these mice in a manner that they gained their immunological competence. The setup employed ensures that the foreign antigens of the graft do not cause central tolerance, so the analysis gives information about peripheral events. Anderson found that grafts with multiple minor histocompatibility differences were rejected, whereas those with only one were not immunogenic but induced tolerance.^35^, ^36^ Anderson argues that the rejection of the grafts with multiple histocompatibility differences can be explained by the Quorum Hypothesis and shows the Historical Postulate to be invalid.^2^

The observations made in AIRE mice, and Anderson’s own studies,^33^, ^35^, ^36^ that I refer to as the Anderson study, have contributed to Anderson’s conclusion that The Quorum Hypothesis is valid; if the number of lymphocytes generated within a given time exceeds quorum, CD4 T cells are activated by antigen; if not, antigen inactivates the CD4 T cells. This better explains the facts than the postulate, as it can readily account for the fact that there is a limitation on the foreignness of a peripheral antigen such that it induces tolerance.

My view is different when faced with the same observations. I am struck by the fact that, though *several experimental reports* show that the postulate does not hold for some antigens in different situations, *it holds, as far as we can tell, for all peripheral self‐antigens under normal physiological circumstances*. The Historical Postulate is pertinent to how *self‐antigens* achieve tolerance, whether centrally or peripherally. What is different about these ‘exceptional’ experimental situations from the situation of natural peripheral self‐antigens? These antigens are more foreign than naturally occurring peripheral antigens, as the Anderson study so beautifully leads one to infer.^35^, ^36^ As the postulate holds for *natural peripheral antigens under normal physiological circumstances*, I say it is valid, in the context of peripheral tolerance. One cannot expect this postulate to hold outside the physiological limits that existed when ‘the postulate was exploited’, by evolution, to realize the attribute of peripheral self‐nonself discrimination. This is why biological laws are often more circumscribed than physical laws.

Why are the studies with TcR transgenic mice regarded by Anderson as not seriously violating the postulate, whereas he thinks the autoimmune immune responses seen in the AIRE^−/−^ mice and his own observations violate it? In the case of AIRE^−/−^ mice, the rate of generation of mature T cells specific for peripheral antigens is greater than in wild‐type mice. I suggest that in this case, and in Anderson’s study, the rate is sufficiently great, so quorum is achieved before lymphocytes specific for some peripheral antigens are irreversibly inactivated, and so responses against these peripheral antigens are generated. I believe Anderson agrees with this view. I will make the case in the next section that we, and our immune system, live on the edge: it is not surprising that slight perturbations upset the balance between tolerance and immunity. (Anderson reasonably says the studies involving TcR transgenic mice are much further removed from normal physiology than the system employed in his own studies. I agree. That makes the observations of his study a greater challenge to understand.)

I must state in passing and for clarity that I believe Anderson and I agree with the facts and their interpretation at the molecular and cellular levels. We disagree with how they are best presented at the level of the system. Anderson suggests that, in the case of peripheral tolerance, The Historical Postulate is clearly not valid, whereas the Quorum Hypothesis is sufficient to provide a mechanism of peripheral tolerance and accounts for diverse observations. I like to express the situation by stating that The Historical Postulate is valid within certain limits, and that peripheral antigens are antigens that are within these limits.

Indeed, the initial reports of antigens unable to induce peripheral tolerance were surprising to me. One of the earliest studies was that of Anderson and Matzinger.^33^ They showed that immunodeficient female mice reject established male skin grafts when the immune system is generated. This and other studies led to me realize there are limitations on the mechanism of peripheral tolerance. On reflection, I realized I should have anticipated these limitations. I came to realize the biological significance of these findings when thinking about the evolution of peripheral tolerance, as I discuss in greater detail below.

I would like, though, to first illustrate why I think differently from Anderson concerning these ‘exceptions’ to the postulate. Suppose we found an antigen of a parasite that expressed a PAMP that interacted with a PPR to upregulate the CoS molecule on APC required for CD4 T‐cell activation. This antigen could and would activate single, antigen‐specific CD4 T cells. Suppose this antigen is employed in similar studies as Anderson employed to test The Historical Postulate: does this antigen induce peripheral tolerance if present before and as the immune system is generated? From all we know, tolerance would not be established but rather immunity. This antigen can activate a single CD4 T‐cell specific for the antigen as it emigrates from the thymus. What would we conclude? Suppose we came to understand that immunity was generated because this antigen bore the particular PAMP it does, with the properties outlined above. I would say that these observations do not violate the postulate, as this is a very particular case; moreover, no peripheral antigens bear such a PAMP and so the postulate holds *for all peripheral self‐antigens*. Furthermore, the study shows how important it is that peripheral self‐antigens do not express such PAMPs. I similarly suggest it would be unreasonable to use the AIRE^−/−^ and Anderson’s studies as evidence against the postulate; rather they show that there is a limit on how foreign peripheral antigens can be if peripheral tolerance is to be achieved.

It might seem that, as we understand how peripheral tolerance is achieved in terms of The Quorum Hypothesis, the question of whether the broader postulate is valid is really of little consequence. I would make two general points in response to this thought. Firstly, any more detailed mechanism, such as The Quorum Hypothesis, consistent with a broader level postulate, such as the Historical Postulate, makes the broader level postulate in some sense redundant; it has served its use in guiding the development of a proposal for a more detailed mechanism. In addition, a postulate at the level of the system can be pertinent to several detailed mechanisms. The considerations we have been discussing relate to peripheral tolerance. The Historical Postulate is pertinent to central tolerance, to peripheral tolerance in the sense just explored, and in other situations. For example, Matzinger suggests her Danger Model, a competing model *at the level of the system* for what determines whether antigen activates or ablates CD4 T cells, can explain tolerance to new antigens that occur at the time of puberty. I will argue below that this is implausible; my considerations as to how tolerance to such ‘adolescent antigens’ *may* be achieved is guided by the postulate.

Another use of the postulate is to focus attention on some self‐antigens for which the postulate *cannot* apply. As I shall discuss later, the idiotypes of the receptors of newly generated B cells and T cells may constitute such antigens, as they ‘appear’ after the first lymphocytes are generated. In this case, the postulate is valuable in drawing attention to a special situation of interest. This is another means by which ideas at the level of the system can guide our more detailed considerations. I suggest that postulates at the level of the system should not be regarded as absolute statements, as just illustrated by the ‘PAMP‐expressing peripheral self‐antigen’, but as a guide to more detailed mechanisms. I suggest later how it might be that the immune system is most often ignorant of or tolerant of these idiotypes. This potential tolerance does not in my mind show the postulate to be wrong, as the postulate applies to most central and peripheral self‐antigens, it is just not complete.

## 10 CONSTRAINTS ON QUORUM SIZE AND IMPLEMENTATION TIMES INVOLVED IN THE ACTIVATION AND INACTIVATION OF CD4 T CELLS

Considerations/observations at the level of the system both guide and integrate considerations and the interpretation of observations at more detailed levels. As argued above, a conflict between more detailed observations with considerations or observations at the level of the system show that at least one of them to be wrong; alternatively, the exception to the general statement might reflect a situation outside normal physiological limits. I think the observations on AIRE^−/−^ mice are highly interesting in this respect. I suggest they illustrate how tightly related and tuned different processes are. I have found it enlightening to consider the evolutionary, selective forces on certain processes in two different animals, ones in which AIRE does not operate, and ones in which AIRE does. I suggest that these considerations give an insight into the advantages to an animal where AIRE operates, over its counterpart that has evolved in the absence of AIRE. Such considerations also bring to the fore the interdependence of central and peripheral mechanisms of tolerance, why AIRE^−/−^ mice are autoimmune, and how different temporal parameters governing the activation and the inactivation of lymphocytes are related and of critical importance.

Consider a vertebrate without natural AIRE expression of ‘peripheral antigens’ in primary lymphoid organs. We assume peripheral tolerance is achieved via the process incorporated in The Quorum Hypothesis. What evolutionary forces govern the size of the quorum? Clearly, the prime reason for a quorum requirement for lymphocyte activation is to ensure that single or a few CD4 T cells specific for a peripheral antigen are not activated but are inactivated by peripheral self‐antigens. The rate at which lymphocytes specific for a peripheral self‐antigen is generated and emigrate from primary lymphoid organs depends in this case on the degree to which they are ‘uniquely foreign’. We assume that, in animals without AIRE, there is no central tolerance to the ‘peripheral antigens’. The degree to which they are ‘uniquely foreign’ will depend upon the size of the antigen and the degree to which its epitopes are unique and not shared with other self‐antigens, against which self‐tolerance is centrally established. (The nature of the lymphocyte repertoire will also be a factor, but for simplicity we suppose this to be constant in the different hypothetical animals we consider, and so can be ignored.) The rate of generation of lymphocytes specific for different peripheral antigens will be different. Consider the peripheral antigen, G, for which this rate is the greatest. If reliable tolerance is to be established against G, the quorum will need to be relatively higher than if it were to guarantee tolerance to a ‘less foreign’ peripheral self‐antigen. Thus, guaranteeing tolerance to G means the size of the quorum that nature can tolerate must be a certain, minimal size. However, the larger the quorum size, the slower are immune responses generated against foreign invaders. Speed is of the essence in defence, and so ways of decreasing the number of G‐specific lymphocytes that emigrate from primary lymphoid organs will be evolutionarily favoured, allowing the quorum to be smaller, and so responses against invaders faster.

Thus, in an animal that naturally does not express peripheral antigens in primary lymphoid organs by an AIRE‐dependent mechanism, the speed of immune responses against *all* foreign invaders would appear to be limited by requiring a relatively high quorum number to ensure reliable tolerance to G. Given that an AIRE‐like mechanism evolved, there would be different evolutionary pressures to express different ‘peripheral antigens’ in primary lymphoid organs; the greatest pressure would be to express ‘peripheral antigens’, such as G, to decrease the rate at which G‐specific lymphocytes emigrate from primary lymphoid organs. This would allow the quorum size to be decreased and still allow peripheral tolerance to be maintained and, importantly, to increase the speed of responses against foreign invaders.

These considerations provide a context for understanding the evolutionary, selective pressures that operate to control various parameters governing interdependent processes. I believe they illustrate how system‐level considerations inform the consideration of mechanisms at a more detailed level. I do not think the autoimmune phenotype of AIRE^−/−^ mice provide grounds for discarding The Historical Postulate. Rather, I believe the ‘limits’ on the postulate allowed me, at least, to better appreciate the evolutionary context within which the postulate is realized.

### 10.1 The dependence of tolerance or rejection on the site of the graft

Anderson and Matzinger have shown that male *skin grafts* on immune‐incompetent females are rejected as immune competence is established.^35^ In contrast, male grafts to internal sites, such as under the kidney capsule of immune‐incompetent females, are not only accepted as the immune system is established, but the mice become tolerant to the male antigen.^36^ How to explain this in the context of The Historical Postulate? The question of why the site of implantation may be so important was raised by Anderson.^2^ It led me to certain considerations.

As explained above, there is a dialectic between the processes leading to the activation and inactivation of CD4 T cells. The smaller the quorum, the less likely is peripheral tolerance to be reliably established, but the more rapid can immune responses be to foreign invaders. Given that the skin is a first, primary barrier against many invaders, it seems natural to me that there is an armada of innate defence mechanisms in skin compared to internal sites, such as under the kidney capsule. I suggest that evolution might well have resulted in different circumstances obtaining at skin and internal grafting sites, resulting in adaptive immunity being intrinsically more favoured than tolerance for skin grafts than for grafts at internal body sites. The most extreme possibility would be that evolution has ensured there are no skin‐specific peripheral antigens, and so no need for peripheral tolerance of skin antigens! In this case quorum could be very small indeed for skin grafts, as governed by different properties of some cells in the pertinent draining nodes, as compared to lymph nodes draining other sites, without any obvious pathological consequences. This example illustrates how the size of the quorum at different sites of the body may have evolved to be different in a manner that both realizes tolerance overall and provides the most effective defence against invaders.

### 10.2 The danger model and the historical postulate

Anderson quotes me: ‘the main contemporary model of how lymphocytes are activated vs. inactivated by antigen, the Danger model, is inconsistent with the historical postulate’. He continues: ‘To some degree this is setting up a strawman to be torn down’.^2^ Anderson suggests that, as danger signals are only rarely generated, most lymphocytes specific for peripheral antigens will be deleted. This is in accord with the idea that the continuous presence of self‐antigens results in unresponsiveness. I beg to disagree. I think it obscures significant considerations worthy of examination.

I have in my mind two potential versions of the Danger Model: where DAMP signals are only required to activate naïve CD4 T cells, and where DAMP signals are periodically required to sustain the on‐going activation of CD4 T cells. I am uncomfortable with the plausibility of both possibilities. Anderson has made it clear in his comments as a referee that Matzinger espouses the latter possibility.^8^ Nevertheless, given the importance of these issues, I think it worthwhile to consider both possibilities, and for me to express why I am uneasy with both.

Consider first the case where a DAMP signal is only required to activate a naïve CD4 T cell. Primary immune responses are not that rare. The difficulty with this scenario, as far as I am concerned, is this: once an anti‐self CD4 T cell is activated, no further control on the activation of its descendants is envisaged, and so CD4 T clonal expansion of anti‐self CD4 T cells can occur as well as that of CD4 T cells specific for foreign antigens that are persistently present, as often occurs following many infections. It would be important in this scenario to prevent the activation of naïve CD4 T cells specific for peripheral self‐antigens as effectively as possible. Whether a newly arising anti‐self CD4 T cell is activated is envisaged to depend upon whether a danger signal is present, and this in turn is envisaged *not* to depend upon whether the immune system has been exposed to the antigen in the past, as envisaged in The Historical Postulate, and as incorporated in The Quorum Hypothesis. It is in this sense that I consider The Danger Hypothesis to be incompatible with The Historical Postulate. The Danger Hypothesis provides insufficient discrimination, between the activation of newly arising CD4T cells specific for peripheral self‐antigens and those specific for foreign antigens, to be plausible to me. I might consider the danger idea, in accordance with Sherlock Holmes' dictum that the unlikely must be true when no other possibility exists, if the dictum held. However, I can envisage a more discriminatory means by which peripheral tolerance of CD4 T cells can be achieved. The virtue of the quorum idea,^23^ proposed a decade or two before Janeway’s pathogen‐associated molecular pattern (PAMP)^5^ and the Danger Hypothesis,^7^ is that it provides better discrimination of self from nonself and is also consistent with The Historical Postulate. I should note that it appears that even a population of activated CD4 T cells can be inactivated by antigen if quorum cannot be met.^37^, ^38^, ^39^ This may allow the population of anti‐self CD4 T cells, resulting from the activation of rare anti‐self CD4 T cells by a foreign antigen that cross‐reacts with a peripheral self‐antigen, to be inactivated by the self‐antigen, when and if the cross‐reacting antigen sufficiently dissipates.^37^

The second, alternative version of The Danger Hypothesis, that I now appreciate Matzinger favours, is that the sustained activation by antigen, of a CD4 T cell population, requires the periodic stimulation by a danger signal.^8^ The virtue of this possibility is that it would provide further checkpoints on the sustained activation of anti‐self CD4 T cells. However, as adult animals/people have many on‐going immune responses in secondary lymphoid organs, including those to hidden/partially hidden PAMP‐free self‐antigens,^28^, ^29^ the generation of danger signals in this case would be virtually ubiquitous and, as such signals are not antigen‐specific, would likely undermine peripheral tolerance. A potential response by a danger proponent could be that danger signals are highly localized. Second signals are best focused if initiated by recognition by lymphocytes of antigen, and delivered by a linked mechanism, as in The Quorum Hypothesis.

Anderson suggests the Danger Model is consistent in large measure with the postulate, as danger signals are rare and so lymphocytes specific for peripheral self‐antigens will usually be eliminated as generated. I hope the immediately preceding paragraphs express my unease with this view.

### 10.3 Revisiting Janeway’s thoughts

A major argument put forward by Janeway, in favour of his PAMP model, is that it is difficult to raise immunity to many purified protein antigens, unless they are given in an adjuvant, such as CFA, that contains microbial products containing PAMPs.^5^ Matzinger has also invoked similar considerations.^8^ Janeway referred to this fact as ‘the immunologists' dirty little secret’. These antigens were most often foreign, vertebrate proteins, homologous to self‐antigens of the immunized host. Such antigens were chosen just because they were not very immunogenic. This allowed investigators to examine whether these antigens were paralytic under any circumstances, that is, were able to generate unresponsiveness. It is very difficult to generate unresponsiveness with highly immunogenic antigens. Using antigens unable to generate strong immune responses also allowed investigators to determine what manipulations, such as coupling them to a foreign carrier, were required to result in them becoming more immunogenic. Such manipulations obviously provided clues as to what was required to generate an immune response. These weakly immunogenic antigens were for the most part homologous, foreign vertebrate proteins, and so generally less foreign than non‐PAMP expressing bacterial antigens, of similar size; their poor immunogenicity is understandable in terms of The Quorum Hypothesis. More complex, vertebrate antigens, such as foreign red cells, are highly immunogenic without adjuvant. Thus, there are more plausible explanations than Janeway’s for understanding the basis of ‘the immunologists’ dirty little secret’.

### 10.4 Late‐appearing antigens

Anderson considered, in his explanation of The Danger Model, the case of ‘late‐appearing’ self‐antigens, and how The Danger Model could explain tolerance to these antigens.^1^ Indeed, the case of late appearing antigens seems to have been a major consideration in Matzinger’s formulation of The Danger Model.^7^, ^8^ I feel the plausibility of The Historical Postulate has influenced my thinking on the subject of ‘late‐appearing’ antigens.

I suggest the existence of ‘late‐appearing antigens’ may be much less frequent than sometimes assumed. An animal has the genes for virtually all potentially ‘late‐appearing’ antigens. It seems to me, as one who finds The Historical Postulate attractive as a means of ensuring reliable tolerance, that these antigens may be expressed earlier in other sites of the body than where they first ‘appear’ to appear. There is a simple test of this idea. Consider a supposedly ‘late‐appearing antigen’, believed to be first expressed at puberty, a situation invoked by Matzinger.^7^, ^8^ In this case, it should be easier to raise immunity to the antigen in an individual who has not yet reached puberty than in a more mature individual. This expectation is somewhat analogous to the finding that tadpoles can be immunized to make antibodies against frog haemoglobin.^40^ I am not aware that this type of study has been successful. It seems to me more plausible that nature has solved the problem of ‘late‐appearing’ antigens by an ‘AIRE‐like’ mechanism that is in accord with The Historical Postulate, that is, their earlier expression at other sites that their appearance at ‘late’ sites.

### 10.5 An interesting case of late appearing‐antigens: idiotypic epitopes

I was concerned, when first thinking about the quorum idea well over 50 years ago, that there was a certain class of ‘late‐appearing’ self‐antigens that must be considered. Tolerance against these antigens could not be explained by The Historical Postulate. It is plausible, I believe, that most self‐antigens are first expressed before lymphocytes are generated, and so tolerance towards them can be achieved. There is one obvious exception. These are antigens unique to particular lymphocytes. Obviously, such antigens cannot exist before lymphocytes have been generated. The idiotypic epitopes of antibodies were then the most obvious example of this type of antigen. The ideas I had then, and their relationship to pertinent and published observations, have only been briefly alluded to before. They seem particularly pertinent to this article and to be of some broader interest.

Immunization with antibody/antigen complexes can be employed to raise rheumatoid factor, and antibodies that recognized epitopes present on the Fc regions of antibodies in antibody–antigen complexes but not on free antibody.^41^ Antibodies to idiotypic epitopes are readily generated by immunizing with the idiotypic antibody complexed with the *foreign antigen* that the antibodies are specific for.^41^ In both cases, it seemed likely that the ‘foreign antigen’ is acting as a carrier in a manner understandable in the context of the quorum idea. My thoughts were that idiotypes on antibodies not complexed with antigen, and produced by the progeny of recently activated B cells, might have very few new epitopes and so would be paralytic, if produced in sufficient amounts.

I read in 1968 a review by Potter and Lieberman on allotypes and idiotype.^42^ They had a Table there that showed one needed, to get an anti‐idiotypic antibody response, an allotype difference between the antibody bearing the idiotype and the antibodies of the immunized recipient. These observations were made in studies immunizing with antibody not complexed with antigen. The observations then led me to think the idiotypic epitopes of the myeloma protein would be paralytic in the absence of a foreign, allotypic epitope that could act as a carrier.

Iverson subsequently published two letters in Nature that supported these thoughts. These showed that a myeloma protein was immunogenic for both idiotype and allotype, when the allotype is foreign in the immunized mouse; when there is no allotype difference, the idiotypic epitope of the antibody is immunogenic only if the mice are primed to a hapten and the mouse challenged with the haptenated myeloma protein.^43^ This is a case where a hapten appears to act as a carrier! The administration of the unhaptenated myeloma protein resulted in paralysis of idiotypic‐specific B cells.^44^ These observations surely fit in with the quorum idea. The relevance of these observation to idiotypes expressed by the Ig receptors of B cells is less clear, as the antibody as a membrane receptor is in such a different physical form from free antibody. Nevertheless, it may well be that the critical step in preventing anti‐idiotypic immune responses to newly arising B cells in most cases is the inactivation of T helper cells specific for idiotypic epitopes. The antibody is likely minimally foreign as so many related antibodies would have been produced in large amounts, rendering the individual tolerant to most of the epitopes present on any ‘new’ antibody. These considerations are pertinent in considering the intrinsic plausibility/implausibility of Jerne’s Idiotype Network Theory.^45^ The idea that there is generally an internal reactivity between lymphocytes belonging to the same immune system, resulting in their mutual activation, rather than reactivity being directed primarily against foreign invaders, always seemed to me to be both implausible and unattractive.^46^

## Data Availability

Data sharing is not applicable to this article as no new data were created or analyzed in this study.
